# Functional Analysis of Ficolin-3 Mediated Complement Activation

**DOI:** 10.1371/journal.pone.0015443

**Published:** 2010-11-10

**Authors:** Estrid Hein, Christian Honoré, Mikkel-Ole Skjoedt, Lea Munthe-Fog, Tina Hummelshøj, Peter Garred

**Affiliations:** Laboratory of Molecular Medicine, Department of Clinical Immunology, Rigshospitalet, Faculty of Health Sciences, University of Copenhagen, Copenhagen, Denmark; University of California Merced, United States of America

## Abstract

The recognition molecules of the lectin complement pathway are mannose-binding lectin and Ficolin -1, -2 and -3. Recently deficiency of Ficolin-3 was found to be associated with life threatening infections. Thus, we aimed to develop a functional method based on the ELISA platform for evaluating Ficolin-3 mediated complement activation that could be applicable for research and clinical use. Bovine serum albumin (BSA) was acetylated (acBSA) and chosen as a solid phase ligand for Ficolins in microtiter wells. Binding of Ficolins on acBSA was evaluated, as was functional complement activation assessed by C4, C3 and terminal complement complex (TCC) deposition. Serum Ficolin-3 bound to acBSA in a calcium dependent manner, while only minimal binding of Ficolin-2 and no binding of Ficolin-1 were observed. No binding to normal BSA was seen for any of the Ficolins. Serum C4, C3 and TCC deposition on acBSA were dependent only on Ficolin-3 in appropriate serum dilutions. Deposition of down stream complement components correlated highly significantly with the serum concentration of Ficolin-3 but not with Ficolin-2 in healthy donors. To make the assay robust for clinical use a chemical compound was applied to the samples that inhibited interference from the classical pathway due to the presence of anti-BSA antibodies in some sera. We describe a novel functional method for measuring complement activation mediated by Ficolin-3 in human serum up to the formation of TCC. The assay provides the possibility to diagnose functional and genetic defects of Ficolin-3 and down stream components in the lectin complement pathway.

## Introduction

The complement system is an integral part of the innate immune system that protects the host against invading pathogens. Three distinct pathways constitute the complement system; the classical pathway, the alternative pathway and the lectin pathway [1]. The C1 complex initiates the classical pathway upon recognition of immune complexes and dying host cells [2]. The alternative pathway is spontaneously activated by C3 hydrolysis, but it has also been reported that properdin, a stabilizer of the alternative pathway convertase [3], is capable of initiating the complement cascade [4]. The Ficolins and mannose-binding lectin (MBL) in association with MBL/Ficolin-associated serine proteases (MASPs) are the initiator molecules of the lectin pathway. Three MASPs (−1, −2 and −3) have been described so far and the current notion is that MASP-2 is the main lectin pathway activator. Upon recognition of pathogen-associated molecular patterns or altered self by MBL and the Ficolins, the associated proteases cleave C4 and C2, hereby activating the complement cascade which ultimately leads to the formation of the TCC [5].

Deficiencies in the initiator molecules of the complement system have resulted in a more profound understanding of the role of the three pathways in the innate immune system. Individuals deficient in the C1 complex, C4 and to a lesser degree C2 are likely to suffer from severe bacterial infections and systemic lupus erythematosus (SLE) [6]. Lack of factor D results in defect alternative pathway activation and is associated with *Neisserial meningitidis* infections [7]. Mutations in the properdin gene resulting in complete absence of properdin or expression of non-functional protein are also associated with meningococcal disease [8], emphasizing the role of the alternative pathway in the defence against meningococci. Polymorphisms in both the promoter and coding region of the MBL gene (MBL2) have been shown to influence the concentration and function of expressed MBL, resulting in deficiency [9]. Approximately 10–15% of the Caucasian population are functional MBL deficient, making this the most common deficiency in the complement system. In most cases, MBL deficient individuals appear healthy; however, during certain settings MBL deficiency is associated with increased risk for diseases and disease outcome [5].

The knowledge regarding the association between the Ficolins and disease is scarce and comes primarily from association studies of single nucleotide polymorphisms (SNPs) [10]. Several SNPs have been described in the *FCN1* gene encoding Ficolin-1 (also referred to as M-Ficolin) [11]. Two of these SNPs, one located in the promoter and one in exon 9 of the *FCN1* gene have been associated with susceptibility to rheumatoid arthritis [12]. SNPs in the promoter and coding region of the *FCN2* gene are associated with concentration and function of the protein [13], [14]. A few association studies between *FCN2* SNPs and Ficolin-2 (also referred to as L-Ficolin) serum levels have been published [15]–[18], however more studies are needed to clarify the role of Ficolin-2 in disease. Several polymorphisms have been identified in the promoter region of the gene (*FCN3*) encoding Ficolin-3 (also referred to as H-Ficolin), but it appears that none of these are associated with the varying levels found in serum [19]. However, a frame shift mutation in exon 5 (*FCN3 + 1637delC*) of the *FCN3* gene, leading to a premature termination of transcription was shown to be associated with lower levels of Ficolin-3 in the heterozygous state [19]. Ficolin-3 has been suggested to participate in the clearance of dying host cells [20], [21]. We have recently described a patient with a history of severe recurrent infections that was homozygous for the *FCN3+1637delC* mutation and had no detectable Ficolin-3 in serum [22], suggesting that complete lack of Ficolin-3 is a novel immunodeficiency associated with disease.

The involvement of the complement system in various diseases has stirred the development of several assays to evaluate deficiencies in the three activation pathways. Screening for complement deficiencies are now a days often conducted by functional assays based on enzyme-linked immunosorbent assay (ELISA) techniques for evaluating the classical, the alternative and the MBL-mediated lectin pathway [23]–[25]. To our knowledge, no assay that can assess complement activation mediated specifically by the Ficolins in serum has been developed. Furthermore, the high frequency of individuals deficient in MBL makes it difficult to evaluate deficiencies in down stream lectin pathway components such as MASP-2 using the currently existing functional assays.

Here we describe the development of an ELISA based assay for the evaluation of Ficolin-3 mediated complement activation, that will enable the detection of deficiencies and functional defects in Ficolin-3 and its associated proteases. This assay will provide a critical addition to the current functional assays used for evaluating complement deficiencies.

## Results

**Figure 1:**
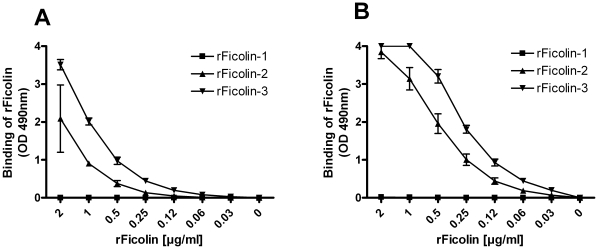
Binding of recombinant Ficolins to acBSA. Microtiter plates were coated with acBSA and subsequently incubated with equal concentrations of rFicolin-1 (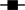), rFicolin-2 (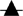), rFicolin-3 (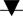) at (**A**) 30 min at 37°C or (**B**) 3 h at RT. Binding was detected with monoclonal antibodies directed against the individual proteins. Graphs show mean ± SD (n = 4).

In order to assess the ability of acBSA to function as a ligand for human Ficolins, we applied recombinant Ficolins in known concentrations to microtiter wells coated with acBSA and assessed the binding of the individual Ficolins using monoclonal antibodies. When plates were incubated for 30 min at 37°C, we detected binding of both rFicolin-2 and rFicolin-3, while no binding of rFicolin-1 was seen (Figure 1A). Increasing the incubation time to 3 h at room temperature resulted in a greater binding of both rFicolin-2 and rFicolin-3 and again, no binding of rFicolin-1 was detected (Figure 1B). At both incubation times, rFicolin-3 appeared to bind better than rFicolin-2.

**Figure 2:**
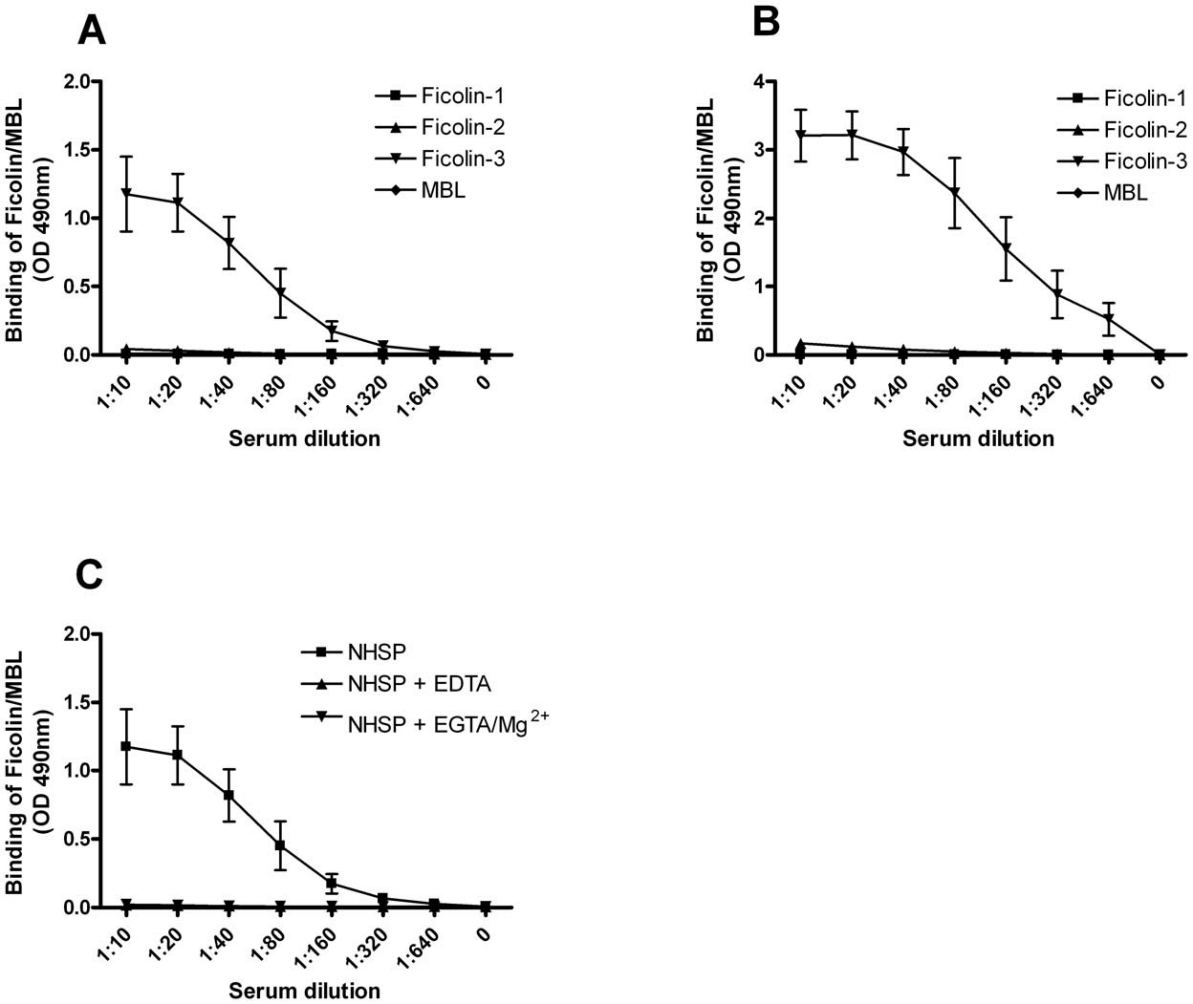
Binding of serum Ficolins and MBL to acBSA. Microtiter plates were coated with acBSA and subsequently incubated with a NHSP for either (**A**) 30 min at 37°C or (**B**) 3 h at RT. Binding of Ficolin-1 (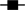), Ficolin-2 (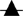), Ficolin-3 (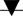) or MBL (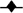) was subsequently detected with monoclonal antibodies directed against the individual proteins. (**C**) acBSA incubated with: NHSP (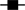), NHSP with 10 mM EDTA (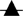) or NHSP with 10 mM EGTA and 5 mM Mg^2+^ (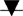). Binding was detected with a monoclonal antibody against Ficolin-3. All graphs show mean ± SD (n = 3).

Next, we evaluated the binding of Ficolins and MBL from human serum to acBSA. Immobilized acBSA was incubated with a normal human serum pool (NHSP) and binding of the individual Ficolins and MBL was detected by monoclonal antibodies. When immobilized acBSA was incubated with serum for 30 min at 37°C, only binding of Ficolin-3 was seen, while no Ficolin-1, Ficolin-2 or MBL binding could be detected (Figure 2A). Incubating the plates with serum for 3 h at room temperature resulted in a profound increase in the binding of Ficolin-3 compared to the plate incubated for 30 min at 37°C (Figure 2B). Furthermore, a low binding of Ficolin-2 could be detected, while no Ficolin-1 or MBL binding was seen (Figure 2B).

Ficolin-3 has been shown to contain a calcium-binding site in the proximity of the ligand-binding site [26], suggesting that calcium is required for Ficolin-3 binding to ligands. We examined this by chelating calcium and magnesium from serum using EDTA or calcium alone by EGTA with addition of Mg^2+^. Binding of serum Ficolin-3 to acBSA was completely abrogated in both settings (Figure 2C), demonstrating that Ficolin-3 interacts with acBSA in a calcium dependent manner.

**Figure 3:**
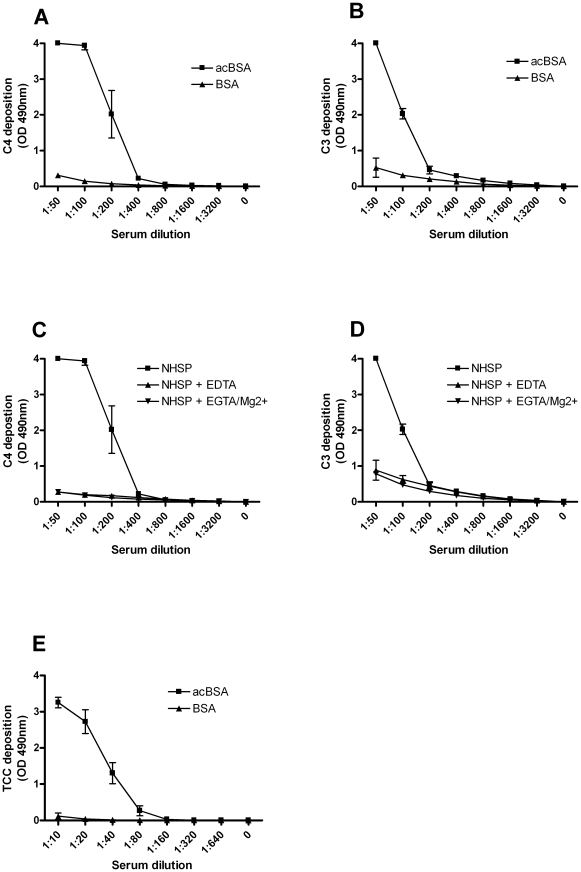
Complement deposition of C4, C3 and TCC on acBSA. (**A–B**) Microtiter wells were coated with acBSA or BSA and subsequently incubated with NHSP for 30 min at 37°C. (**A**) C4 and (**B**) C3 deposition on acBSA (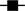) or BSA (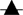) was detected using a polyclonal antibody. (**C–D**) Microtiter wells were coated with acBSA and subsequently incubated with NHSP (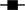), NHSP with 10 mM EDTA (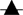) or NHSP with 10 mM EGTA and 5 mM Mg^2+^ (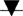) for 30 min at 37°C. (**C**) C4 or (**D**) C3 deposition was detected as described above. (**E**) Finally, microtiter wells were coated with acBSA (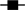) or BSA (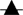) and subsequently incubated with NHSP in serial dilution for 45 min at 37°C and TCC-deposition on acBSA was detected using a monoclonal antibody. All graphs show mean ± SD (n = 4).

In order to test whether the binding of Ficolins to acBSA resulted in complement activation we coated microtiter wells with either untreated BSA or acBSA and subsequently incubated with a NHSP. Deposition of both C4 and C3 was observed in the wells coated with acBSA, while only very small amounts of deposition were seen in the BSA-coated wells incubated with the highest serum concentrations (Figure 3A–B). Deposition of C4 and C3 on acBSA was almost completely abrogated with the addition of EDTA or EGTA + Mg^2+^ to the NHSP (Figure 3C–D), demonstrating that complement activation on acBSA is calcium dependent. Detection of TCC was obtained when the incubation time of serum was increased to 45 min at 37°C. The result was dose dependent deposition of TCC on acBSA where as no activation was observed on BSA (Figure 3E).

**Figure 4:**
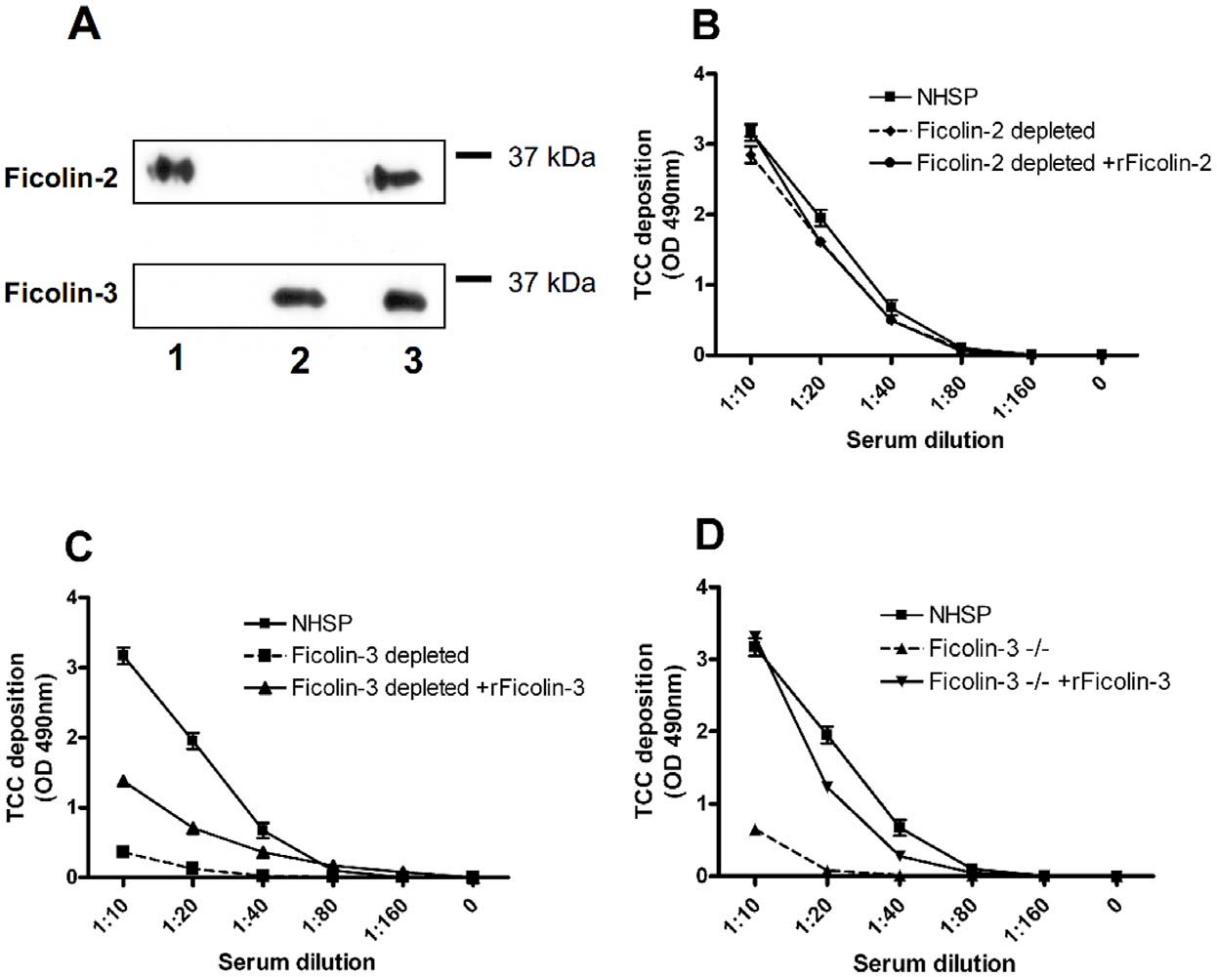
TCC deposition on acBSA with depleted and deficient sera. Ficolin-2 or Ficolin-3 was depleted from serum using specific monoclonal antibodies. (**A**) Depleted serum was applied to SDS-PAGE/Western blotting and probed for Ficolin-2 or Ficolin-3. Lane 1: Ficolin-3 depleted serum. Lane 2: Ficolin-2 depleted serum. Lane 3: Control depleted serum. (**B–D**) Microtiter plates were coated with acBSA and incubated for 45 min at 37°C with different sera before TCC deposition was detected with a monoclonal antibody. (**B**) Shows TCC deposition for: NHSP (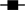), Ficolin-2 depleted serum (-♦-), Ficolin-2 depleted serum with addition of 5 µg/ml rFicolin-2 (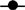). (**C**) Shows TCC deposition for: NHSP (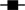), Ficolin-3 depleted serum (-▪-) and Ficolin-3 depleted serum with addition of 25 µg/ml rFicolin-3 (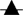). (**D**) Shows TCC deposition for: NHSP (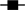), Ficolin-3 deficient serum −/− (-▴-), Ficolin-3 deficient serum −/− with addition of 25 µg/ml rFicolin-3 (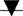). All graphs show mean ± SD of duplicate wells.

Our binding studies indicate that it is primarily serum Ficolin-3 that binds to acBSA under the experimental conditions used (Figure 2). To further examine whether the complement activation observed on acBSA was exclusively mediated by Ficolin-3 we depleted serum of either Ficolin-2 or Ficolin-3 using specific antibodies or a non-related isotype matched antibody as a control. Depletion of Ficolin-2 and Ficolin-3 from serum was confirmed by SDS-PAGE and Western blotting (Figure 4A). We then applied the Ficolin-2 depleted, Ficolin-3 depleted and control serum to immobilized acBSA and evaluated the C4 (Data S1), C3 (Data S2) and TCC deposition (Figure 4B–C). Depletion of Ficolin-2 produced no obvious difference in complement deposition compared to NHSP (Figure 4B). Furthermore, addition of 5 µg/ml rFicolin-2 to the Ficolin-2 depleted serum did not show any influence on the complement deposition. When adding Ficolin-3 depleted serum to the plates however, the complement deposition was remarkably reduced (Figure 4C). Spiking the Ficolin-3 depleted serum with 25 µg/ml rFicolin-3 resulted in a reconstitution of complement activation substantially, but not to the same level as in non-depleted serum (Figure 4C). Serum from a Ficolin-3 deficient individual (−/−) previously described [22] was also evaluated and showed only low complement deposition in the highest serum concentration in level with the Ficolin-3 depleted serum (Figure 4D). Reconstituting the deficient serum with rFicolin-3 increased the deposition of complement components almost to the level of the NHSP deposition curve (Figure 4D).

**Figure 5:**
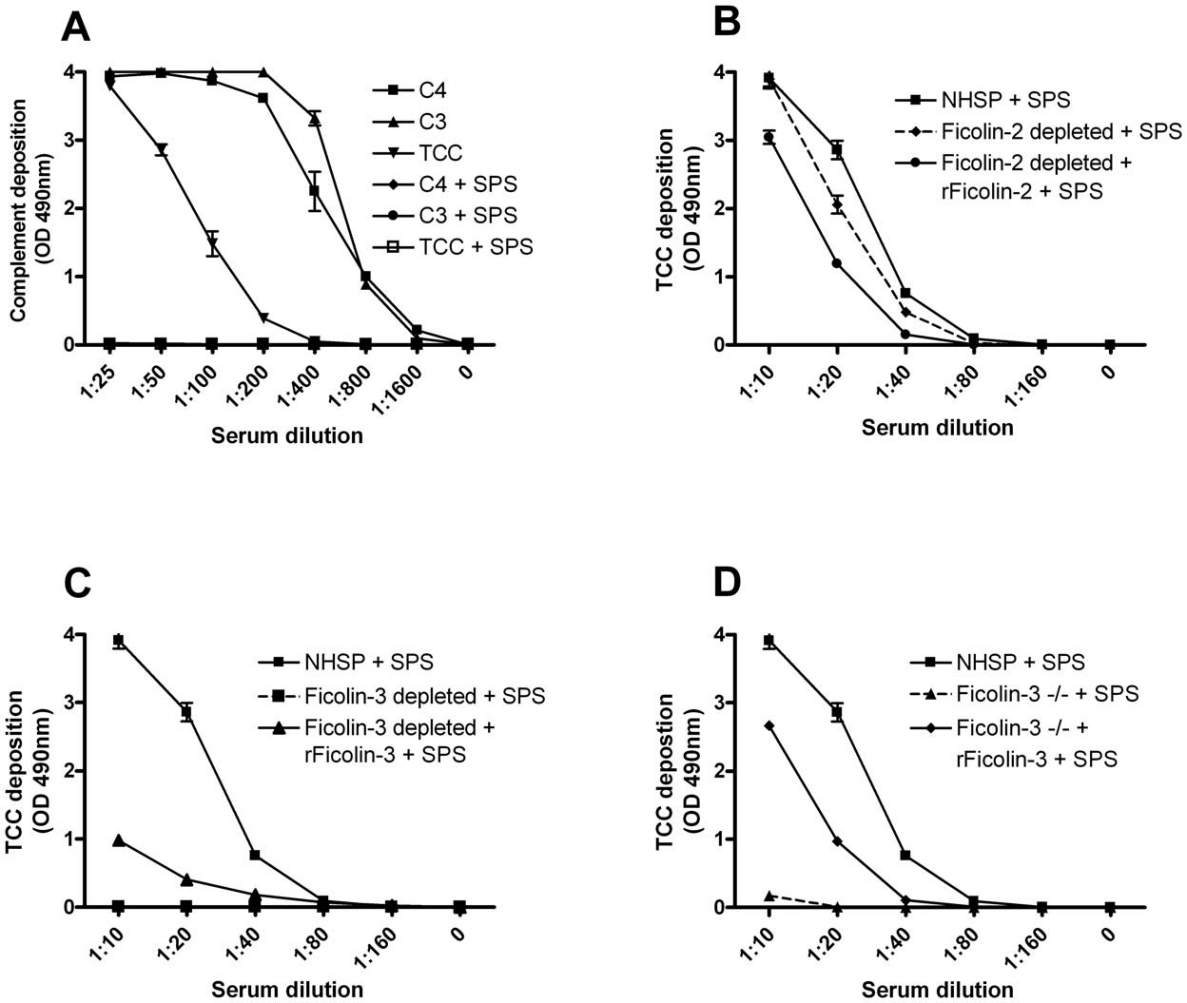
Inhibition of the classical pathway of complement activation with SPS. (**A**) Classical pathway activation was obtained by coating microtiter wells with HSA and subsequently incubated with a rabbit-anti-HSA antibody. A NHSP was pre-incubated with or without SPS for 5 min on ice and then applied to the microtiter wells for 30 min (45 min for TCC-detection) at 37°C. Complement activation was detected with biotinylated antibodies. In the absence of SPS: C4 (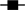), C3 (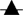) or TCC (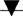); and in the presence of SPS: C4 (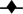), C3 (-•-) or TCC (-□-). (**B–D**) Different sera pre-incubated on ice with SPS and then incubated for 45 min at 37°C in microtiter plates coated with acBSA before TCC deposition was detected with a monoclonal antibody. (**B**) Shows TCC deposition in the presence of SPS for: NHSP (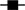), Ficolin-2 depleted serum (- ♦ -), Ficolin-2 depleted serum with addition of 5 µg/ml rFicolin-2 (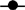). (**C**) Shows TCC deposition in the presence of SPS for: NHSP (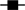), Ficolin-3 depleted serum (-▪-) and Ficolin-3 depleted serum with addition of 25 µg/ml rFicolin-3 (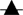). (**D**) Shows TCC deposition in the presence of SPS for: NHSP (-▪-), Ficolin-3 deficient serum −/− (-▴-), Ficolin-3 deficient serum −/− with addition of 25 µg/ml rFicolin-3 (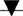). All graphs show mean ± SD of duplicate wells.

Individual serum samples could contain anti-bovine antibodies [27] that potentially could mediate complement activation on acBSA via the classical pathway and interfere with the interpretation of the results. It has recently been shown that sodium polyanethole sulfonate (SPS) within a concentration window inhibits complement activation via the classical and the alternative pathway but does not influence the lectin pathway [28]. To test whether this compound indeed inhibits classical complement pathway activation on albumin we coated microtiter plates with human serum albumin (HSA) and rabbit-anti-HSA IgG to create solid phase immune complexes. Serum pre-incubated with 0.5 mg/ml SPS before added to the wells and when we subsequently evaluated C4, C3 and TCC deposition, SPS was able to completely inhibit complement deposition of all the components (Figure 5A), consistent with previous findings [28]. To evaluate the effects of adding SPS to serum in the Ficolin-3 assay based on acBSA we employed different sera: NHSP, NHSP depleted of Ficolin-2 or Ficolin-3, respectively, NHSP depleted and then reconstituted with rFicolin-2 or rFicolin-3, respectively, and finally serum from the Ficolin-3 deficient individual (−/−) with or without addition of rFicolin-3 and evaluated the C4 (Data S1), C3 (Data S2) and the TCC deposition (Figure 5B–D). Addition of SPS to normal serum and Ficolin-2 depleted serum revealed no striking change in complement deposition (compare Figure 4B with Figure 5B). Furthermore, reconstituting the Ficolin-2 depleted serum with rFicolin-2 did not generate a difference compared to serum with no rFicolin-2 application with or without SPS. When evaluating Ficolin-3 depleted or Ficolin-3 deficient serum a residual complement deposition of TCC was seen in high serum concentration (Figure 4C–D), however, when adding SPS to these sera, the remaining activity was reduced to base line levels - the only exception being a minimal activity from the Ficolin-3 deficient serum (Figure 5C–D). Finally, the minimal complement deposition observed on untreated BSA in low serum dilutions was eliminated when adding SPS (Data S3).

**Figure 6:**
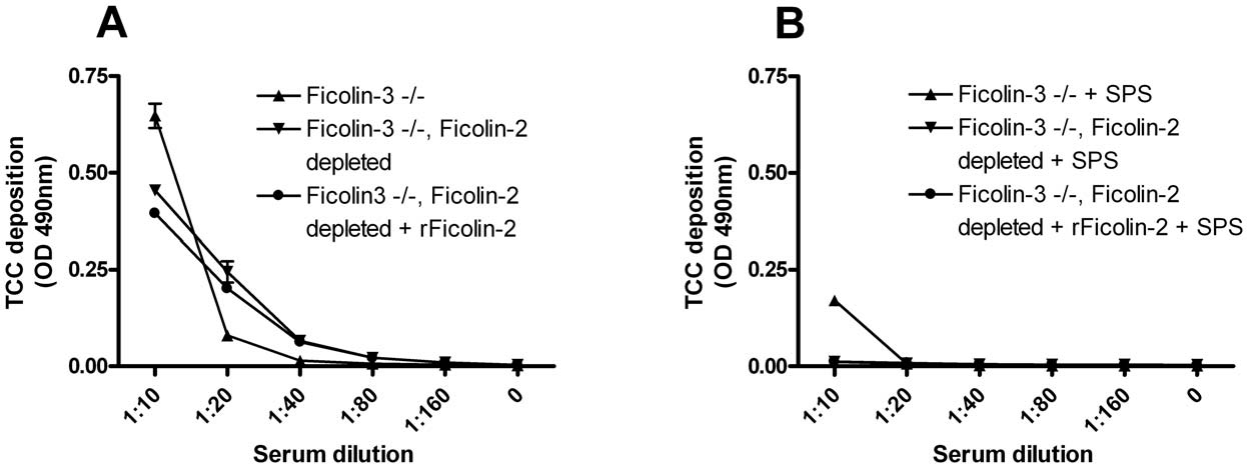
TCC deposition on acBSA with Ficolin-3 deficient serum depleted of Ficolin-2 without and with SPS. Serum pre-incubated on ice (**A**) without SPS or (**B**) with SPS and then incubated for 45 min at 37°C on microtiter plates coated with acBSA. The sera analysed was: Ficolin-3 deficient serum −/− (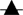), Ficolin-3 deficient serum −/− depleted of Ficolin-2 (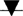), Ficolin-3 deficient serum −/− depleted of Ficolin-2 with addition of 5 µg/ml rFicolin-2 (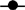). TCC deposition was detected with a monoclonal antibody. Graphs show mean ± SD of duplicate wells.

Since the residual complement deposition in presence of SPS was only seen in serum from the Ficolin-3 deficient individual and not in the Ficolin-3 depleted serum, we wanted to examine this further. Hence, we depleted the Ficolin-3 deficient serum of Ficolin-2 and added rFicolin-2. The different sera were evaluated for TCC deposition with and without SPS. The residual activity was eradicated when the Ficolin-3 deficient serum was depleted of Ficolin-2 and evaluated in the presence of SPS (compare Figure 6A–B). Again, the addition of rFicolin-2 had no influence on the complement activity.

**Figure 7:**
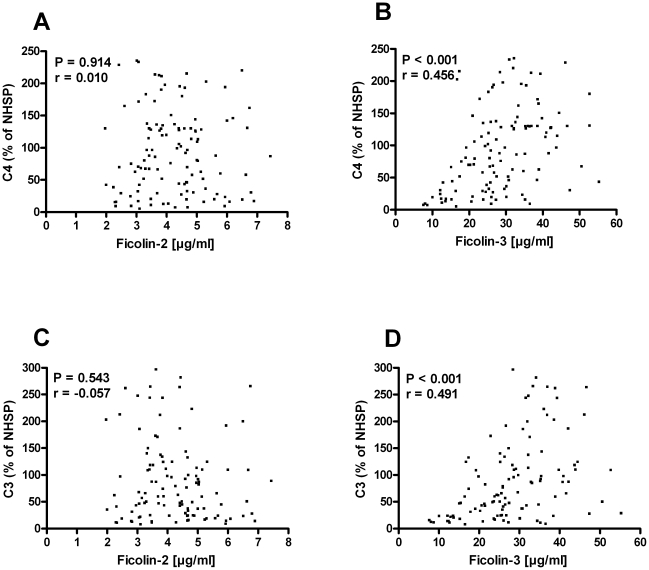
Correlation of C4 and C3 deposition on acBSA to Ficolin-2 and -3 serum concentrations. Microtiter wells were coated with acBSA and subsequently incubated with individual serum samples in triplicates for 30 min at 37°C. The relative deposition of C4 and C3 on acBSA from serum from 116 individuals was determined using a NHSP as standard (100%). Ficolin-2 and Ficolin-3 concentrations in serum were determined as described in materials and methods. The graphs show the deposition of C4 as a function of (**A**) Ficolin-2 or (**B**) Ficolin-3 serum concentrations and the deposition of C3 as a function of the (**C**) Ficolin-2 and (**D**) Ficolin-3 serum concentrations. P-values and Spearman rank (r) were calculated using the GraphPad Prism software. (n = 116).

Serum concentrations of Ficolin-2 and -3 in 116 blood donors were determined. Ficolin-1 was not evaluated since a specific assay for determining the serum concentration of Ficolin-1 was not available to us. We then evaluated the C4 and C3 deposition on acBSA from serum from the 116 individuals. No obvious correlation of the Ficolin-2 serum concentration to either C4 (Figure 7A) or C3 (Figure 7C) deposition was observed. In contrast, the deposition of both C4 (Figure 7B) and C3 (Figure 7D) showed a significant positive correlation with the serum concentration of Ficolin-3.

**Figure 8:**
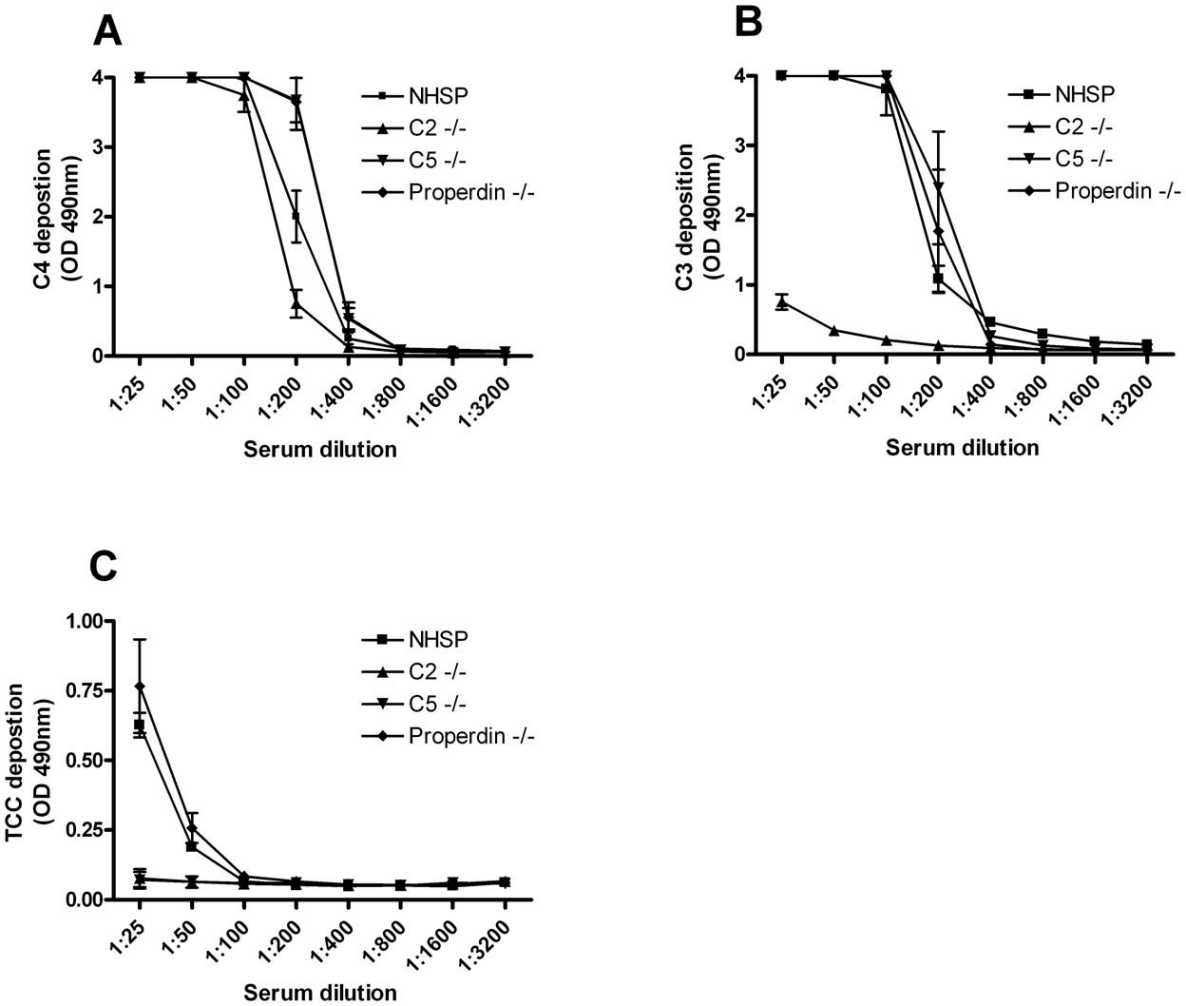
Complement activation on acBSA using C2, C5 or properdin deficient serum. Microtiter plates were coated with acBSA and subsequently incubated with NHSP (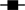), C2 deficient serum (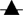), C5 deficient serum (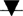) or properdin deficient serum (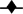) in serial dilutions for 30 min at 37°C. (**A**) C4 deposition and (**B**) C3 deposition on acBSA measured using polyclonal antibodies. (**C**) TCC deposition on acBSA measured using a monoclonal antibody. All graphs show mean ± SD (n = 3).

Finally, in order to establish whether this assay could detect deficiencies in downstream components of the Ficolin-3 mediated complement activation, we assessed the C4, C3 and TCC deposition on acBSA using C2, C5 and properdin deficient sera, respectively. All three sera mediated C4 deposition on acBSA (Figure 8A). Both the C5 and properdin deficient sera mediated C3 deposition at levels comparable to the serum pool that was used as a control (Figure 8B). However, the C3 deposition was almost completely abrogated when the C2 deficient serum was applied (Figure 8B). Finally, when assessing the TCC, both the C2 and C5 deficient serum failed to mediate deposition on acBSA, while the properdin deficient serum showed normal deposition of the TCC (Figure 8C).

## Discussion

Deficiencies in the complement system are often associated with recurrent infections and autoimmune diseases [6]. We have recently described a patient suffering from recurrent infections and obstructive lung disease that was homozygous for the *FCN3 + 1637delC* mutation, resulting in complete deficiency of Ficolin-3 [22]. The allele frequency of the *FCN3 + 1637delC* mutation is approximately 0.01 in the Danish population [19]. Thus, one would expect that 1 out of 10.000 Caucasian individuals would be homozygous for the *FCN3 + 1637delC* mutation. The likely association between Ficolin-3 deficiency and disease was the primary motivation for this report, since we sought to develop an assay that could assess complement activation mediated specifically by Ficolin-3. Such assay could aid the identification of individuals with functional and genetic deficiencies of Ficolin-3 and perhaps also serve as a useful diagnostic tool in collaboration with the existing assays for evaluating complement function.

Acetylated compounds have previously been shown to function as ligands for the Ficolins [29]–[31]. However, the observation that serum deficient of Ficolin-3 did not mediate C4 deposition on acBSA [22] prompted us to investigate whether this ligand would be suitable for assessing Ficolin-3 mediated complement activation. The observed binding of rFicolin-2 was in accordance with previous studies [30], however it was of interest that rFicolin-3 appeared to bind even better than rFicolin-2. The rFicolin-1 used for this study displayed the characteristic oligomeric pattern when applied to SDS-PAGE/Western blotting under non-reducing conditions and was fully functional in another binding assay [32]. Thus, the specific assay conditions applied in this assay are the likely explanation for the lack of binding of rFicolin-1. Interestingly, when evaluating the binding of serum Ficolins, we only observed binding of Ficolin-3 to acBSA when incubated with serum for 30 min at 37°C. One plausible explanation for this could be the difference in serum concentration of Ficolin-2 and Ficolin-3. Ficolin-2 circulates at a mean concentration of 5.4 µg/ml [14], while Ficolin-3 circulates at an approximately five-fold higher mean concentration [19]. However, when evaluating binding of the recombinant Ficolins added in equal concentrations, rFicolin-3 still appeared to have a better binding capacity to acBSA at the applied assay conditions compared to rFicolin-2.

Based on the initial binding results of serum Ficolins to acBSA, we decided to proceed with the evaluation of C4 and C3 deposition on acBSA using the 30-minute, 37°C serum incubation step, since this appeared to provide the properties for Ficolin-3 binding to acBSA without interference from Ficolin-2. The complement deposition on acBSA yielded a serum dose dependent curve and only a small amount of C3 deposition on untreated BSA was evident in the highest serum concentration. This could be a result of passive absorption from serum, or the marginal generation of active C3-convertases. These results demonstrate that the complement deposition in intermediate serum dilutions is specific for acetylated BSA and not untreated BSA.

The binding site of Ficolin-3 to ligands is through the fibrinogen-like domain and the crystal structure of Ficolin-3 has shown that a calcium-binding site is located in the vicinity of the binding site, suggesting that calcium might be required for Ficolin-3 binding to its ligand [26]. In agreement with this, we observed that the binding of serum Ficolin-3 to acBSA was completely absent in the presence of EDTA and EGTA/Mg^2+^ as were the deposition of complement components C4 and C3.

Evaluating depleted sera showed, that Ficolin-2 does not contribute substantially to complement deposition in this assay. Adding rFicolin-3 to Ficolin-3 depleted serum, complement activation on acBSA was reconstituted, though not to the level of full serum. A reasonable explanation for this is that when serum is depleted of Ficolin-3, a certain amount of MASPs are also depleted since Ficolin-3 circulates in complex with the MASPs. Hence, addition of rFicolin to the depleted serum reconstitutes some complement activation; however, the MASPs will still remain as a limiting factor for complement activation. The Ficolin-3 deficient serum described by Munthe-Fog *et al.* [22] showed the same absence of complement deposition on acBSA as the Ficolin-3 depleted serum. Reconstitution of complement activation with rFicolin-3 allowed full restoration of complement activity. This observation substantiates the explanation that MASPs are removed from serum along with Ficolin-3 in the Ficolin-3 depleted serum. The level of MASPs in the Ficolin-3 deficient serum is unaffected and may thus not be a limiting factor of complement activation in this serum as in the Ficolin-3 depleted serum.

We did not deplete serum of Ficolin-1, since we did not have any specific antibodies against this protein. However, considering the low concentration of Ficolin-1 in serum [33], [34] and the observation that rFicolin-1 did not interact with acBSA, it appears likely that Ficolin-1 does not mediate complement activation at the applied assay conditions. Moreover, it has been shown that the complement activating potential of Ficolin-1 is very low compared with that for Ficolin-2 and Ficolin-3 [35].

We did not observe any binding of serum MBL to acBSA, suggesting that MBL does not influence the complement activation on acBSA. This is in agreement with previous results, which demonstrated that MBL defect serum mediated C4 deposition on acBSA at levels comparable to a NHSP [22]. Furthermore, we have previously shown that C1q deficient serum is capable of depositing C4 on acBSA at levels comparable to normal human serum, suggesting that the complement activation occurs independently of the classical pathway [22]. However, interference from the classical pathway on acBSA could potentially occur in serum containing anti-bovine antibodies [27].

A recent study has shown that sodium polyanethole sulfonate (SPS) within a certain concentration window inhibits complement activation via both the classical and the alternative pathway and leaves the lectin pathway unaffected [28]. Here, we demonstrate that SPS can inhibit classical pathway activation without significantly influencing complement activation from normal human serum on acBSA. On the other hand, deposition of complement on untreated BSA was abolished in the presence of SPS. When evaluating complement deposition on acBSA with Ficolin-3 deficient or depleted serum, residual complement activation in high serum concentrations seen without SPS was abolished in the presence of SPS. However, even with SPS the Ficolin-3 deficient serum still showed a minimal complement activating activity in the highest serum concentration. We speculated whether this could be due to a minor contribution of complement activation from Ficolin-2 according to the binding experiments. Hence, in an individual with Ficolin-3 deficiency, Ficolin-2 would probably under optimized conditions be able to exhibit some binding to acBSA with the lack of competition from Ficolin-3 in serum. Furthermore, the equilibrium between the MASPs, and the pattern recognition molecules of the lectin pathway will be skewed towards Ficolin-2 in the Ficolin-3 deficient serum and therefore could increase the complement activation potential of Ficolin-2. Contrarily, in the case of the Ficolin-3 depleted serum, a certain amount of MASPs will be removed from serum in complex with Ficolin-3 in the action of depletion. Consequently, the remaining Ficolin-2 would lack endogenous MASPs for complement activation.

To further investigate the possible contribution of Ficolin-2 in this assay, we depleted Ficolin-3 deficient serum of Ficolin-2 and evaluated it in the presence of SPS. We ascribe the residual activity from this serum only seen in the highest serum concentrations to Ficolin-2, which could also fit with the results of the binding experiments. Nevertheless, in diluted serum this potential problem seems to disappear.

The positive correlation between Ficolin-3 concentration and complement activation in 116 serum samples, further substantiates our observations that complement activation on immobilized acBSA under the specific assay conditions employed is primarily mediated by Ficolin-3 and not Ficolin-2, which displayed no correlation. In the correlation plot of Ficolin-3 to C4 and C3 deposition several samples are observed with a rather high serum concentration of Ficolin-3 but yet a relatively low complement activation value. This is likely due to individuals with lowered levels of components further down the complement cascade such as MASP-2, C4 or C2, which will skew the level of complement activation. It should be noted that the inhibitor SPS was not added to the assay buffer when measuring the donor samples, which to some extend could blur the Ficolin-3 results. However, it would be obvious to utilize SPS in the final assay.

In order to address whether the Ficolin-3 mediated complement activation on acBSA would detect deficiencies in downstream complement components, we used sera deficient in C2, C5 and properdin, respectively [36], [37]. C4 deposition was observed at comparable levels to the control NHSP for the C2 deficient serum, while the C3 and the TCC deposition were completely absent. This demonstrates that the formation of a C4bC2a convertase is required for complement activation on acBSA, suggesting that there is no involvement of the alternative pathway. In agreement with this, the properdin deficient serum was fully capable of activating complement on acBSA. The different levels of C4 and to a lesser degree C3 deposition from the individual sera are most likely a result of inter-individual differences in the Ficolin-3 concentrations or other complement components. Taken together, these results demonstrate that the Ficolin-3 mediated complement activation assay is suitable to detect deficiencies in downstream components of the complement cascade.

Homozygosity for an amino acid substitution in MASP-2 (D120G) is causing very low levels of MASP-2 and deficiency in lectin pathway-mediated complement activation [38]. MASP-2 deficiency due to D120G homozygosity has been reported in few patients suffering from multiple infections and/or autoimmune symptoms [38]–[40], but also in apparently unaffected individuals [41], suggesting that MASP-2 deficiency is not necessarily associated with disease, but might function as a disease modifier. Although not formally tested in this study, it is likely that Ficolin-3 mediated complement activation on acBSA will also detect MASP-2 deficiency. The high frequency of MBL deficiency and functional defects [5], [9] makes MBL mediated complement function assays unsuitable for elucidation of deficiencies downstream of MBL itself. The described Ficolin-3 deficiency would occur in approximately 1 out of 10.000 Caucasian individuals [19], [22], making the Ficolin-3 mediated complement activation much more suitable for detection of deficiencies of components downstream of the lectin pathway than an MBL dependent assay.

In this assay we have strived towards as physiological conditions as possible concerning incubation temperature and time, pH and ion concentration of the buffer. Since all three Ficolins have been shown to bind to acetylated compounds the importance of keeping the assay conditions under strict surveillance should be stressed. When measuring the C4 and C3 deposition mediated specifically by Ficolin-3, we suggest that the serum is diluted at least 1:100 and incubated for 30 minutes at 37°C. Using an intermediate serum dilution should circumvent interference from the alternative as well as the classical pathway as well as from Ficolin-2; however, since SPS appears to be an excellent inhibitor of classical pathway and also has been reported to inhibit the alternative pathway, we recommend to add this reagent in the serum incubation step according to Palarasah *et al*. [28]. An alternative could be to block the classical pathway with an anti-C1q antibody that has been shown to work well in MBL dependent complement activation assays [24]. In the case of detection of TCC, the serum concentration should be higher, in order to obtain a proper signal. Furthermore, the incubation time should be increased to 45 minutes at 37°C. Again, the use of SPS will circumvent any interference from the other pathways.

Taken together, our results demonstrate that complement activation on immobilized acBSA under the described assay conditions is specifically mediated by Ficolin-3. To our knowledge, this is the first assay that is capable of evaluating complement activation mediated by Ficolin-3 and down stream components and thus will provide a critical supplement to the already existing assays for evaluating complement function and genetic defects.

## Materials and Methods

### Materials

Maxisorp microtiter plates were purchased from NUNC (Roskilde, Denmark). PBS-buffer (10 mM Na_2_HPO_4_, 1.47 mM KH_2_PO_4_, 137 mM NaCl, 2.7 mM KCl, pH = 7.4), Barbital-buffer (4 mM C_8_H_11_N_2_NaO_3_, 145 mM NaCl, 2.6 mM CaCl_2_, 2.1 mM MgCl_2_, pH = 7.4) and 1 M sulphuric acid were all acquired from the hospital pharmacy (Region H Apoteket, Rigshospitalet, Copenhagen, Denmark). Tween-20 was purchased from Merck (Hohenbrunn, Germany). Bovine serum albumin (BSA), human serum albumin (HSA), sodium acetate solution, acetic anhydride and sodium polyanethole sulfonate (SPS) (Cat.no. P2008-5G) were all purchased from Sigma-Aldrich (Copenhagen, Denmark). Rabbit anti-C4c polyclonal antibody, Horseradish-peroxidase (HRP) conjugated rabbit anti-mouse IgG and OPD substrate tablets were purchased from DAKO (Glostrup, Denmark). Rabbit anti-C3c polyclonal antibody was purchased from Dade Behring (Marburg, Germany). Mouse anti-C5b-C9 terminal complement complex (TCC) monoclonal antibody was purchased from Bioporto Diagnostics (Gentofte, Denmark). Horseradish peroxidase (HRP) conjugated rabbit anti-mouse IgG, donkey anti-rabbit IgG and streptavidin were purchased along with nitrocellulose membranes from GE Healthcare (Buckinghamshire, United Kingdom). Magnetic Dynabeads conjugated with anti-mouse IgG antibodies, NuPAGE 10% Bis-Tris gels, 3–8% Tris-acetate gels, MOPS-buffer, Tris-acetate buffer, LDS loading buffer and reducing agent were purchased from Invitrogen (Taastrup, Denmark).

### Recombinant Ficolins (rFicolin)

Recombinant Ficolin-1, -2 and –3 were generated as previously described for rFicolin-2 [42]. Briefly, the Ficolin genes were amplified and tagged with a hexa-histidine sequence in the C-terminal end. The constructs were cloned into expression vectors and transfected into Chinese hamster ovarian cells. His-tagged rFicolin was purified from the supernatant of the cells on a Ni-NTA column (Qiagen, West Sussex, United Kingdom).

### Monoclonal antibodies against Ficolins

Monoclonal antibodies were generated against Ficolin-1 (FCN106), Ficolin-2 (FCN216 and FCN219) and Ficolin-3 (FCN334) and have all been characterized previously [14], [19], [33]. It should be noted that FCN106 shows a slight cross-reactivity with Ficolin-2. A monoclonal antibody against MBL (Hyb-131-10) was purchased from BioPorto Diagnostics (Gentofte, Denmark). Biotinylated polyclonal antibodies against Ficolin-2 and Ficolin-3 were purchased from R&D systems (Abingdon, UK). Biotinylation of antibodies was conducted by incubating 0.5 mg/ml antibody with 100 µg Biotin-N-hydroxysuccinimide ester (Sigma-Aldrich, Copenhagen, Denmark) for 3 h end-over-end at room temperature. Subsequently the antibodies were dialysed against PBS.

### Acetylation of albumin

A 10% dilution (w/v) of BSA in H_2_O was prepared. A total of 500 µl of the 10% albumin solution was mixed with 500 µl of saturated sodium acetate solution (0.74 g/ml). The solution was placed on ice for 1 h and during this period acetic anhydride was added to the solution in 12-minute intervals to a final volume of 50 µl. The acetylated albumin was dialysed against PBS-buffer and the protein concentration was determined by measuring the absorbance at 280 nm.

### Serum samples

Approval for this study was obtained from The Committees on Biomedical Research Ethics of the Capital Region of Denmark. Blood from 116 blood donors was collected with written informed consent in serum glass. Serum was separated from the coagulate by centrifugation (1600× g for 10 min) and subsequently stored at −80°C. A serum pool (NHSP) was established based on thirty randomly selected donors out of the 116 donors above. Ficolin-3 deficient serum was obtained from a patient previously described [22]. C2 and C5 deficient serum was obtained from individuals characterized as previously described [36]. Properdin deficient serum was obtained from an individual previously described [37].

### Serum concentration of Ficolin-2 and Ficolin-3

Concentrations of Ficolin-2 and Ficolin-3 in the established serum pool or individual donors were determined using previously established assays [14], [19]. Briefly, a monoclonal antibody directed against Ficolin-2 (FCN216) or Ficolin-3 (FCN334) was coated onto microtiter plates in PBS overnight at 4°C. Diluted serum samples was applied to the plates and incubated for 3 h. A standard dilution series of pooled human serum with known concentration of Ficolin-2 and Ficolin-3 were added to each assay. Plates were washed and subsequently incubated with a biotinylated Ficolin-2 antibody (FCN219-Biotin) or Ficolin-3 antibody (FCN334-Biotin) overnight at 4°C. HRP-conjugated streptavidin was added to the wells and incubated for 1 h. After washing, OPD substrate solution containing H_2_O_2_ was added and plates were developed for 10–15 min. Addition of 50 µl/well sulphuric acid terminated the enzymatic reaction and the optical density was measured at 490 nm.

### Depletion of Ficolin-2 and Ficolin-3 from serum

Serum from the NHSP was diluted 1:1 in buffer and incubated for 20 min at 4°C with a monoclonal antibody against Ficolin-2 (FCN219), Ficolin-3 (FCN334) or an isotype matched IgG with no human specificity as a control. Following incubation, magnetic dynabeads conjugated with anti-mouse IgG antibodies (Dynabeads Pan Mouse IgG), was added to the serum samples and incubated end-over-end for 30 min. The tubes were then placed on a magnetic rag and the serum supernatant was removed. Incubation with antibodies and beads was repeated once to ensure full depletion. Depletion was confirmed by SDS-PAGE/western blotting using the NuPAGE system according to manufactures protocols (Invitrogen, Taastrup, Denmark).

### Ficolin binding to acetylated BSA (acBSA)

Microtiter plates were coated with 5 µg/ml acBSA in 100 µl/well volumes in PBS-buffer overnight at 4°C. Plates blocked for 1 h with barbital-buffer containing 0.05% tween (barbital-T) and were subsequently washed thrice in barbital-T. Serum diluted in barbital-T or rFicolins was added to the wells and incubated for 30 min at 37°C or 3 h at room temperature. In order to evaluate whether the binding was dependent on magnesium and calcium 10 mM EDTA or 10 mM EGTA with 5 mM Mg^2+^ was added to the serum. Following incubation, plates were incubated with 1 µg/ml of biotinylated monoclonal antibody against Ficolin-1, Ficolin-2, Ficolin-3 or MBL for 2 h at room temperature, shaking. After washing, HRP-conjugated streptavidin was added for 1 h, room temperature. Plates were washed and developed for 10–15 min with OPD substrate containing H_2_O_2_. Addition of 50 µl/well sulphuric acid terminated the enzymatic reaction. The optical density was measured at 490 nm.

### Ficolin-3 complement activation assay

Microtiter plates were coated with 5 µg/ml acBSA in PBS-buffer overnight at 4°C. Plates were blocked for 1 h with barbital-T and subsequently washed thrice in barbital-T. Serum diluted in barbital-T was added to the wells and incubated for 30 min at 37°C (45 min at 37°C for the detection of TCC). In order to evaluate whether the binding was dependent on calcium 10 mM EDTA or 10 mM EGTA with 5 mM Mg^2+^ was added to the serum. Plates were washed in barbital-T and subsequently incubated with anti-C4c (0.13 µg/ml), anti-C4c-biotin (2 µg/ml), anti-C3c (0.32 µg/ml) or anti-C5b-C9 (TCC) (1 µg/ml) for 2 h, shaking at room temperature. Plates were washed in barbital-T and incubated with HRP-conjugated streptavidin, anti-rabbit or anti-mouse IgG antibodies (all diluted 1:2000 in barbital-T) for 1 h at room temperature, shaking. Plates were washed and developed as described above.

### Classical pathway activation assay

Microtiter plates were coated with 5 µg/ml human serum albumin (HSA) in PBS overnight at 4°C. The plates were washed in barbital-T and incubated with 2 µg/ml polyclonal rabbit anti-HSA IgG for 2 h at room temperature as described by Palarasah *et al.* [28]. Plates were washed as before and incubated with dilutions of serum in barbital-T for 30 min at 37°C. Following incubation, plates were washed and incubated with 2 µg/ml biotinylated anti-C4c or anti-C3c polyclonal antibody, or anti-C5b-9 monoclonal antibody for 2 h at room temperature and subsequently HRP-conjugated streptavidin or HRP-conjugated anti-mouse IgG, respectively, for 1 h at room temperature. Plates were washed and developed as described above. Inhibition of the classical pathway was conducted as previously described by Palarasah *et al.* [28]. In brief, full serum was pre-incubated for 5 min on ice with SPS dissolved in TBS to a final concentration of 0.5 mg/ml. Subsequently, serum with SPS was diluted in barbital-T and added to the microtiter plates in serial dilutions.

### Statistical analysis

All statistical analysis were conducted using GraphPad Prism 4 software (La Jolla, CA, USA).

## Supporting Information

Data S1
**C4 deposition on acBSA with depleted and deficient sera.** Ficolin-2 or Ficolin-3 was depleted from serum using specific monoclonal antibodies. Depleted and deficient sera pre-incubated on ice with or without SPS and were then added to microtiter plates coated with acBSA and incubated for 30 min at 37°C. C4 deposition was detected with a polyclonal antibody. (**A**) Ficolin-2 depleted serum analysed without SPS or (**B**) with SPS: NHSP (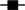), Ficolin-2 depleted serum (-♦-), Ficolin-2 depleted serum with addition of 5 µg/ml rFicolin-2 (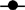). (**C**) Ficolin-3 depleted serum analysed without SPS or (**D**) with SPS: NHSP (-▪-), Ficolin-3 depleted serum (-▪-) and Ficolin-3 depleted serum with addition of 25 µg/ml rFicolin-3 (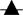). (**E**) Ficolin-3 depleted serum analysed without SPS or (**F**) with SPS: NHSP (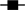), Ficolin-3 deficient serum −/− (-▴-), Ficolin-3 deficient serum −/− with addition of 25 µg/ml rFicolin-3 (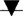). All graphs show mean ± SD of duplicate wells. (TIF)

Data S2
**C3 deposition on acBSA with depleted and deficient sera.** Ficolin-2 or Ficolin-3 was depleted from serum using specific monoclonal antibodies. Depleted and deficient sera pre-incubated on ice with or without SPS and were then added to microtiter plates coated with acBSA and incubated for 30 min at 37°C. C3 deposition was detected with a polyclonal antibody. (**A**) Ficolin-2 depleted serum analysed without SPS or (**B**) with SPS: NHSP (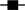), Ficolin-2 depleted serum (-♦-), Ficolin-2 depleted serum with addition of 5 µg/ml rFicolin-2 (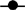). (**C**) Ficolin-3 depleted serum analysed without SPS or (**D**) with SPS: NHSP (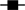), Ficolin-3 depleted serum (-▪-) and Ficolin-3 depleted serum with addition of 25 µg/ml rFicolin-3 (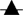). (**E**) Ficolin-3 depleted serum analysed without SPS or (**F**) with SPS: NHSP (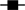), Ficolin-3 deficient serum −/− (-▴-), Ficolin-3 deficient serum −/− with addition of 25 µg/ml rFicolin-3 (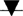). All graphs show mean ± SD of duplicate wells. (TIF)

Data S3
**C4 and C3 deposition on BSA with or without SPS.** A NHSP pre-incubated on ice without SPS (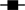) or with SPS (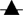) and then incubated for 30 min at 37°C on microtiter plates coated with BSA. (**A**) C4 and (**B**) C3 deposition on untreated BSA was detected with polyclonal antibodies, respectively and (**C**) TCC deposition with a monoclonal antibody. Graphs show mean ± SD of duplicate wells. (TIF)
